# Evolutionary rescue model informs strategies for driving cancer cell populations to extinction

**DOI:** 10.1101/2024.11.26.625315

**Published:** 2024-11-28

**Authors:** Amjad Dabi, Joel S. Brown, Robert A. Gatenby, Corbin D. Jones, Daniel R. Schrider

## Abstract

Cancers exhibit a remarkable ability to develop resistance to a range of treatments, often resulting in relapse following first-line therapies and significantly worse outcomes for subsequent treatments. While our understanding of the mechanisms and dynamics of the emergence of resistance during cancer therapy continues to advance, many questions remain about which treatment strategies can minimize the probability that resistance will evolve, thereby improving long-term patient outcomes. In this study, we present an evolutionary simulation model of a clonal population of cells that can acquire resistance mutations to one or more treatments. We then leverage this model to examine the efficacy of a two-strike “extinction therapy” protocol—in which two treatments are applied sequentially in an effort to first contract the population to a vulnerable state and then push it to extinction—in comparison to that of a combination therapy protocol. We investigate the impact of parameters such as the timing of the switch between the two strikes, the rate of emergence of resistant mutations, the dose of the applied drugs, the presence of cross-resistance, and whether resistance is a binary or a quantitative trait. Our results indicate that the timing of switching from the first to the second strike has a marked effect on the likelihood of driving the population to extinction, and that extinction therapy outperforms combination therapy when cross-resistance is present. We conduct an *in silico* trial that reveals more detailed insight into when and why a second strike will succeed or fail. Finally, we demonstrate that modeling resistance as a quantitative rather than binary trait does not change our overall conclusions.

## Full Text Availability

The license terms selected by the author(s) for this preprint version do not permit archiving in PMC. The full text is available from the preprint server.

